# Supplementary material for: One-Step Synthesis of 3D Graphene Aerogel Supported Pt Nanoparticles as Highly Active Electrocatalysts for Methanol Oxidation Reaction
Source: Nanomaterials (Basel). 2024 Mar 20;14(6):547. doi: 10.3390/nano14060547 (PMC10975690; doi:10.3390/nano14060547)
Supplement: Supplementary file 1 [file nanomaterials-14-00547-s001.zip › nanomaterials-2882099-supplementary.pdf]

Supporting Information

One-Step Synthesis of 3D Graphene Aerogel Supported Pt Nanoparticles as Highly Active Electrocatalysts for Methanol Oxidation Reaction

Figure S1 SEM images of the GO(A), 3DGA(B), Pt/3DGA(C).

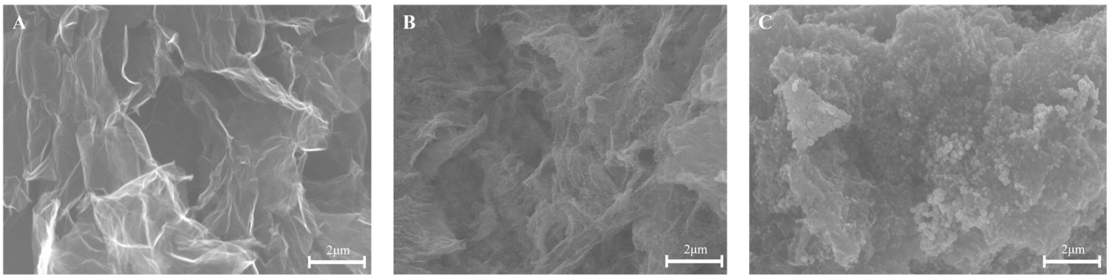

Figure S2 the SEM images of one selected small area and the corresponding EDS results of Pt, C, and O.

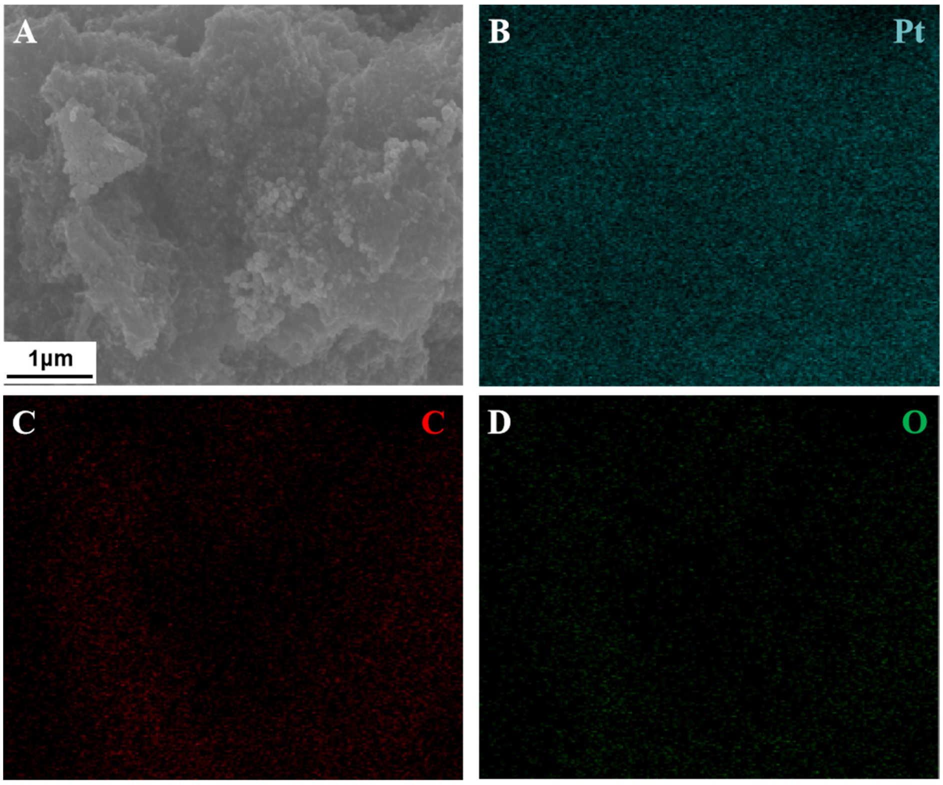

Note S1 Detail information about the content of Pt/graphene aerogel, Pt/carbon black and Pt/graphene

resulting methods	Pt/graphene aerogel	Pt/carbon black	Pt/graphene
Catalyst weight	7.6mg	8.5mg	6.8mg
$\frac{Pt}{Pt+C}$ (%) / XRD	34.1	25.6	29.4

Note S2 Details and the experimental parameter settings.

In order to explore the most suitable reaction temperature and frozen time for preparing Pt/3DGA, 3DGA with the same ratio was prepared at 80, 90, and 100°C. Among them, GA formed poorly at 80°C and appeared jelly-like. While at 100°C, GA did not have a complete 3D network structure. Therefore, we chose 90°C as the reaction temperature

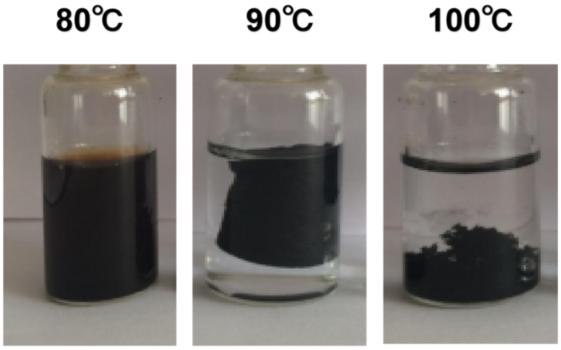

Considering that too short freezing time would make it difficult for the bubbles in the aerogel to transform into ice templates, we selected three freezing times of 6h, 8h and 10h for the test. The results showed that long freezing time would reduce the volume of the original graphene aerogel and made its structure to collapse and deform. Therefore, we believe that choosing 6 hours as the freezing time is the most appropriate.

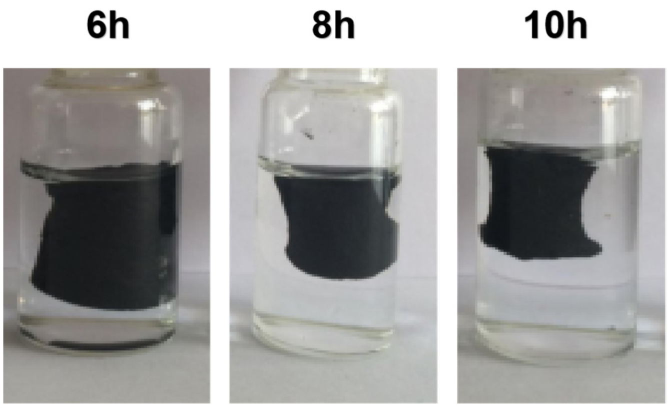

Note S3 The calculation process of electrochemically active surface area（ECSA）

The electrocatalytic performance of Pt/GA, Pt/C and Pt/G in the potential range of -0.2V~1.1V under 1 M H^+ environment was characterized and the electrochemically active surface area (ECSA) of different catalysts were obtained by analyzing of hydrogen adsorption/desorption peaks. The ECSA was calculated from

$$\mathrm{ECSA} = \frac{Q_H}{0.21 \times M_{Pt}}$$

in which Q_H (mC) is the integral charge of the H adsorption peak after calibrating with the baseline of the double layer area and 0.21 mC cm^{-2} is the approximate constant for the adsorption of the single layer H atom from the unit area Pt surface. In addition, M_{Pt} is the Pt mass loaded on the working electrode.
